# Mutagenesis and free energy calculations to optimize the ProRgpB inhibitor loop and identify variants with higher affinity for *Porphyromonas gingivalis* RgpB

**DOI:** 10.1039/d6ra01702a

**Published:** 2026-07-14

**Authors:** Sebastián Tapia, Osvaldo Yañez, Olimpo García-Beltrán, Daniel Bustos, Denisse Bravo, Manuel I. Osorio

**Affiliations:** a Computational & Quantum Enzyme Modeling Lab. Facultad de Odontología, Universidad Andres Bello Santiago, Chile, Echaurren 237 Santiago 8370133 Chile manuel.osorio@unab.cl; b Centro de Modelación Ambiental y Dinámica de Sistemas (CEMADIS), Facultad de Ingeniería y Negocios, Universidad de Las Américas Santiago Chile; c Co-Laboratorio de Investigación en Bioeconomía Regional, Universidad de Ibagué Carrera 22 Calle 67 Ibagué 730002 Colombia; d Centro de Estudios e Investigaciones en Salud y Sociedad, Facultad de Ciencias Médicas, Universidad Bernardo O'Higgins General Gana 1702 Santiago 8370854 Chile; e Facultad de Medicina, Centro de Investigación Biomédica, Universidad Diego Portales Ejército 141 Santiago 8320000 Chile; f Laboratorio de Bioinformática y Química Computacional, Departamento de Medicina Traslacional, Facultad de Medicina, Universidad Católica Del Maule Talca 3480094 Chile dbustos@ucm.cl

## Abstract

Arginine-specific gingipain B (RgpB), a key cysteine protease from *Porphyromonas gingivalis*, is associated with several systemic diseases. It is synthesized as a zymogen bound to a propeptide inhibitor that blocks its catalytic activity until activation in the extracellular environment. To identify inhibitory peptide variants with enhanced affinity, mutants of the propeptide inhibitory loop were designed. A total of 52 mutants were generated and, for each model, four independent molecular dynamics replicas were performed, followed by binding free energy calculations using MM/GBSA. Most variants exhibited more favorable binding affinities than the wild-type (WT) loop (−109.8 ± 4.9 kcal mol^−1^). Among them, V126K (−128.1 ± 5.6 kcal mol^−1^), E131D (−120.6 ± 8.2 kcal mol^−1^), and N132R (−135.8 ± 4.6 kcal mol^−1^) emerged as promising, with the first two showing statistically significant improvements using ANOVA followed by Tukey's post-hoc test (*p* = 0.001). Thermodynamic integration calculations were consistent with increased binding affinity for the selected variants. Thermodynamic integration calculations further confirmed a significant increase in the relative binding affinity for the selected variants compared to the wild type. These results provide a solid basis for future *in vitro* validation and establish an *in silico* framework to accelerate the rational design of propeptide-based inhibitors and the development of novel therapeutic approaches targeting RgpB.

## Abstract 2

Arginine-specific gingipain B (RgpB), a key cysteine protease from *Porphyromonas gingivalis*, is associated with several systemic diseases.

## Graphical abstract

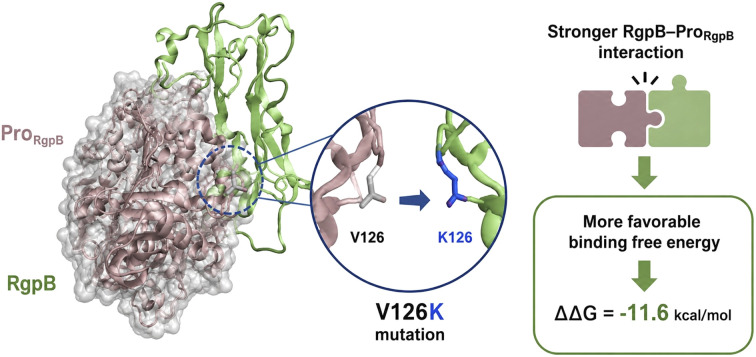

## 1. Introduction

*Porphyromonas gingivalis* (*P. gingivalis*) is a common member of the oral microbiome that can proliferate uncontrollably under dysbiotic conditions.^1^ This overgrowth disrupts the microbial balance and triggers a chronic inflammatory response that extends beyond the oral cavity, reaching other tissues and systems throughout the body.^2,3^ The dysbiosis induced by *P. gingivalis* is associated with both local diseases, such as periodontitis,^2^ and serious systemic pathologies, including Alzheimer's disease,^4–7^ rheumatoid arthritis,^8^ various cardiovascular conditions,^9^ and certain types of cancer.^10,11^ Its ability to invade tissues and evade the immune response largely depends on the gingipains RgpB and Kgp, which are responsible for approximately 85% of the bacterium's total proteolytic activity.^12^ Between the two, RgpB exhibits nearly three times the activity of Kgp.^13^ This central role in bacterial virulence, coupled with its involvement in multiple pathologies, makes RgpB a prime therapeutic target for the development of specific inhibitors.^14^

RgpB is a cysteine peptidase that recognizes arginine residues and shares its catalytic mechanism with several human proteases, making the design of selective inhibitors challenging and increasing the risk of cross-inhibition when substrate-based strategies are used.^15–23^ To overcome this limitation, understanding its regulatory mechanism offers a promising alternative: RgpB is synthesized as a zymogen associated with an inhibitory prodomain (ProRgpB), whose inhibitory loop inserts into the catalytic site, establishing specific interactions that block proteolytic activity prior to secretion.^24^ Upon processing and release in the extracellular environment, the enzyme becomes active, suggesting that the prodomain–enzyme interaction is transient and of moderate affinity.^25^ This opens the possibility of rationally optimizing the prodomain sequence to strengthen these interactions, enhance binding affinity, and develop more selective inhibitors while reducing the risk of cross-inhibition, potentially providing a foundation for future research on the development of highly selective inhibitory strategies.^15–22,24,25^

The inhibition of proteases by zymogenic domains is a real, diverse, and highly specific mechanism, as evidenced in different families such as cathepsin L (I29 domain),^26^ metalloproteinases,^27^*Plasmodium falciparum* subtilisin 1,^28^ and proprotein convertases (PCs).^29^ In the latter, the chimeric PC1/3 variant stands out, achieving nanomolar inhibition and exceptionally high selectivity, highlighting the potential of this approach.^29^ This diversity of interaction modes between the prodomain and the catalytic site provides a solid basis for rational optimization, enabling the design of variants capable of maintaining enzyme inhibition under specific extracellular conditions, such as those found in bodily fluids.^30^ Consequently, these insights are not only applicable to RgpB but could also be extended to the development of selective inhibitory strategies for other proteases that possess zymogenic domains.^26^

The use of propeptides as protease inhibitors represents an innovative approach with high potential, as it exploits intrinsic regulatory interactions unique to each enzyme. However, the marked structural and functional differences among proteases imply that the optimization of this strategy must be addressed on a case-by-case basis, considering both steric and electrostatic complementarity, as well as the mechanism of inhibition.^31^ In the case of the RgpB protease, a promising approach involves the identification of variants of the natural propeptide ProRgpB with higher affinity for the enzyme.^23^ Fine-tuning the region responsible for blocking the catalytic site could lead to the development of inhibitors with high potency and selectivity.

**Fig. 1:**
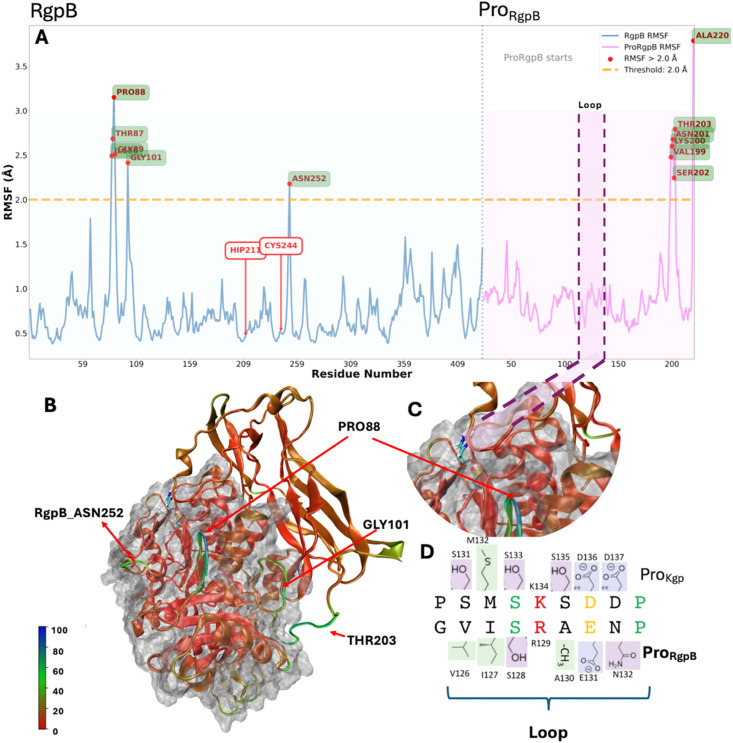
Flexibility of the carbon chain in the RgpB/ProRgpB complex. The root mean square fluctuation (RMSF) of the alpha carbons was analyzed from a 1500 ns molecular dynamics simulation in a box with explicit water. Panel (A) shows the sequence of RgpB in blue and ProRgpB in pink, with red dots highlighting residues with fluctuations greater than 2 Å. In (B), RgpB is highlighted with a gray surface, and the complex structure is colored according to its flexibility, from a maximum of 3.2 Å (blue, 100%) to a minimum (red, 0%), emphasizing residues exceeding 2 Å. Panel (C) shows an increase in the flexibility of the inhibitory loop region, marked with a violet shadow in (A). Finally, (D) presents the sequence of the ProRgpB inhibitory loop compared to that of ProKgp, where conserved residues are shown in green, those with similar chemical properties in yellow, and non-conserved residues in black.

In this context, the inhibitory loop region of ProRgpB shows high sequence conservation with the corresponding region in the Kgp prodomain (ProKgp) (Fig. S1), suggesting a shared inhibition mechanism and enabling a rational design framework.^15^ Comparison of both loops reveals residues that are fully conserved (green), conserved in chemical properties (yellow), and non-conserved (black) (Fig. 1D), guiding substitution strategies: fully conserved residues should be preserved due to their structural and functional roles, residues with similar chemical properties allow conservative substitutions, and non-conserved residues provide greater flexibility for optimization, with the exception of glycine and tryptophan, whose steric and hydrophobic characteristics limit their interchangeability.^32^

Based on this framework, *in silico* mutations can be implemented and, together with molecular dynamics simulations, enable the reproduction of intermolecular interactions and the estimation of the interaction strength between a ligand binding site and the protein.^33^ This approach facilitates the evaluation of the structural and energetic impact of mutations on binding affinity, providing a powerful tool to predict and prioritize variants with improved inhibitory potential prior to experimental validation.^15,31,32,34^

Molecular dynamics (MD) methods are powerful tools for estimating binding free energy variations (Δ*G*) of ligands, including proteins, and assessing affinity changes.^35^ End-point approaches such as MM/GBSA enable rapid, exploratory predictions relative to a reference molecule, although they may overestimate free energy values, whereas more rigorous methods such as alchemical binding free energy (BFE) calculations and the adaptive biasing force (ABF) technique provide higher accuracy and stronger correlation with experimental data. Alchemical methods stand out for their ability to directly estimate relative free energy differences (ΔΔ*G*) between variants with high sensitivity, making them especially suitable for evaluating mutations and optimizing binding affinities.^36^ Although their high computational cost has traditionally limited their use, recent GPU optimizations have significantly reduced these requirements, enabling their practical application in drug discovery. In this context, the present work evaluates the binding affinity of multiple variants through *in silico* mutagenesis guided by residue conservation within the inhibitory domain, followed by molecular dynamics simulations and free energy analyses that combine MM/GBSA and alchemical approaches, establishing a robust computational framework for identifying and prioritizing high-affinity variants for subsequent experimental validation and rational inhibitor design.^33,35,36^

## 2. Results and discussion

### 2.1 Molecular dynamics simulation of the RgpB/ProRgpB complex

To obtain a stable model, a molecular dynamics (MD) simulation of 1.5 µs of the RgpB/ProRgpB complex in a box with explicit water was performed. The complex conformation reached stability after 0.1 µs of simulation, with a root-mean-square deviation (RMSD) below 2.8 Å (Fig. S2). Cluster analysis (Fig. S3) identified a single stable conformation during the last 800 ns of the simulation (Fig. S3A). However, when considering the last 1000 ns (Fig. S3B) or the entire trajectory (Fig. S3C), at least one additional cluster is observed, which likely corresponds to a transient state that had not yet equilibrated, as it is not maintained during the final 800 ns. Analysis of the conformations corresponding to the cluster centroids showed high structural similarity, with differences of less than 2 Å, located in regions distant from both the catalytic site of RgpB and the inhibitory loop of ProRgpB.

**Fig. 2:**
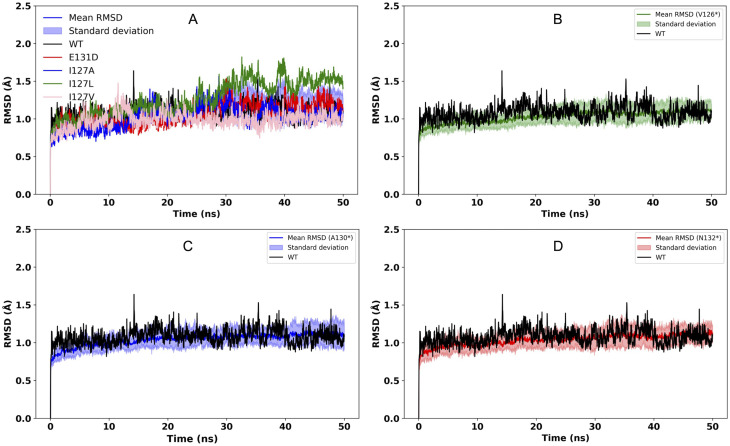
Structural stability of the RgpB/ProRgpB complexes during molecular dynamics simulations. Heavy-atom RMSD values were calculated from 50 ns MD simulations for the WT complex and mutants at positions I127, V126, A130, E131, and N132. Panel A shows the individual RMSD trajectories of the I127 variants (I127A, I127L, and I127V) together with E131D and the WT complex (black line). Panels B–D summarize the behavior of the 18 variants generated at positions V126, A130, and N132, respectively. In these panels, colored lines represent the mean RMSD values, while shaded regions indicate the standard deviation among variants. Specifically, panel B corresponds to the V126* variants (green), panel C to the A130* variants (blue), and panel D to the N132* variants (red).

These findings are consistent with the analysis of the alpha carbon fluctuations (RMSF) of the complex. As seen in Fig. 2A, most of the sequence exhibits RMSF values below 1 Å, confirming the stability achieved. The highest fluctuations (<4 Å) correspond to regions distant from the catalytic site and the inhibitory loop (indicated with red dots in Fig. 2A), which are visualized in green or blue in the complex structure (Fig. 2B). In contrast, the inhibitory loop shows remarkable stability, with RMSF values less than 1.5 Å, highlighted in pink in Fig. 2C.

**Fig. 3:**
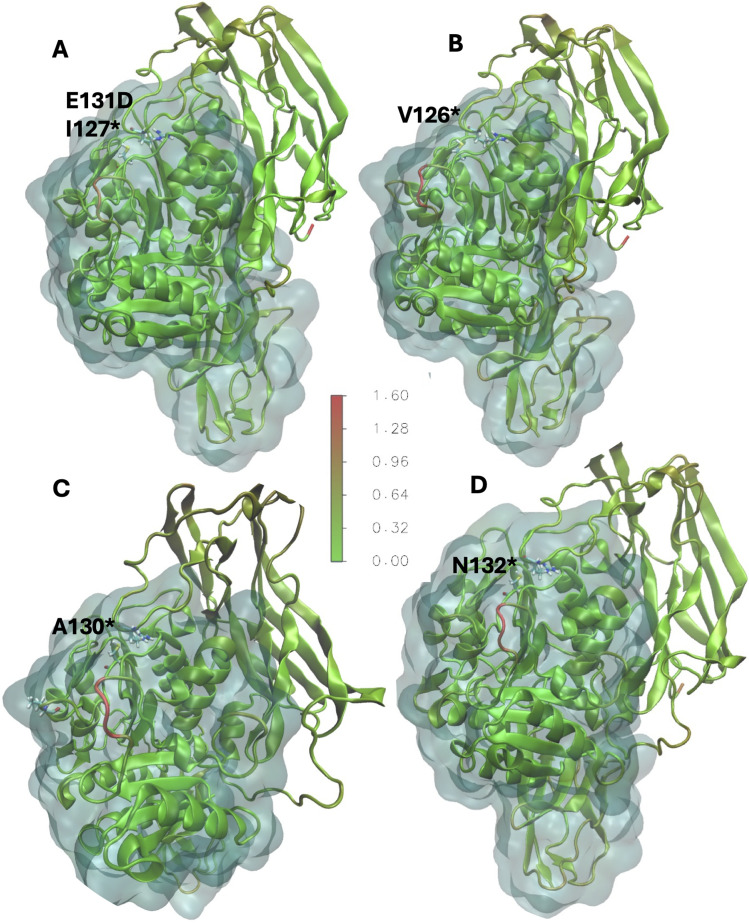
Structural analysis of ProRgpB loop mutants. Superposition of the RgpB/ProRgpB complex structures for the various ProRgpB loop mutants. The coloration of each model represents its root-mean-square deviation (RMSD) after structural alignment with the wild-type (WT) variant.

Considering these structural analyses, which demonstrate the high stability of the complex, the structure corresponding to the centroid obtained from the last 800 ns of the simulation was used for the construction of mutants.

#### 2.1.1 Structural effects of inhibitory loop mutations

Point mutations can increase enzymatic activity or confer greater structural stability under non-physiological conditions. The wild-type enzyme is naturally optimized for the cellular environment and the metabolic needs of the organism, but it can be improved for alternative contexts. Based on this premise, *in silico* mutations were designed in the inhibitory loop of ProRgpB, and the binding affinity of the resulting variants was evaluated using binding free energy calculations. The selection of mutations was based on the degree of residue conservation within the inhibitory domain of RgpB and Kgp, considering the highly conserved residues as essential for the loop's interaction with the catalytic site of RgpB. According to this criterion, loop residues were classified into three categories: fully conserved, conserved in the chemical nature of the side chain, and non-conserved (Fig. 1D). Consequently, fully conserved residues were not modified; those retaining the chemical property were substituted with amino acids of similar characteristics; and for non-conserved residues, all possible substitutions except glycine and tryptophan were tested. This procedure generated a total of 52 modeled mutants. For each one, 50 ns molecular dynamics simulations were performed using AMBER24 with the ff14SB force field,^37^ and binding energies were calculated using the MMGBSA (Molecular Mechanics Generalized Born Surface Area) approximation. The results showed that the variants exhibit stability comparable to the wild-type enzyme (black line in Fig. 2), maintaining RMSD values below 2.5 Å and without showing significant structural deviations throughout the simulation.

**Fig. 4:**
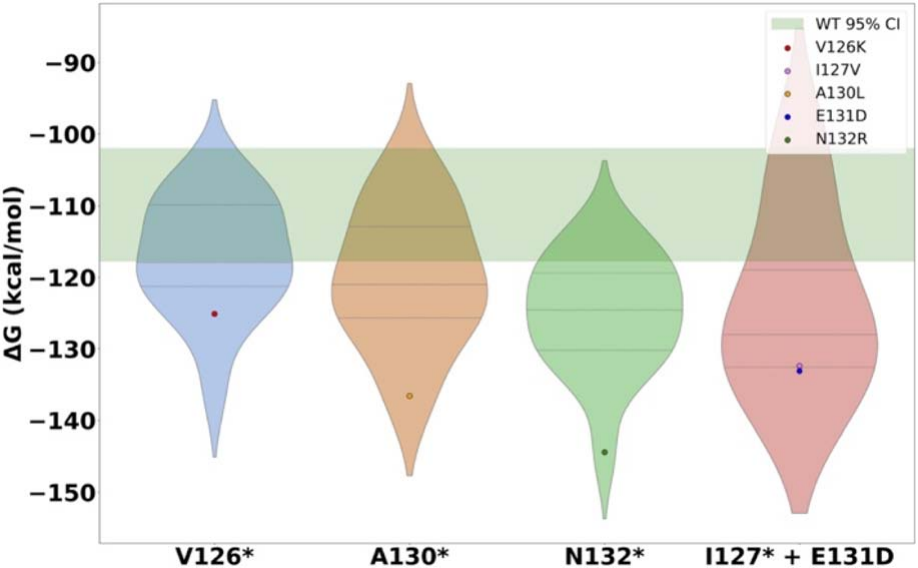
Distribution of Δ*G* (MMGBSA) values for 52 mutants of the ProRgpB inhibitory loop, calculated from 50 ns simulations of *in silico* mutations. A total of 16 mutants were generated for each of the V126* (blue), A130* (orange), and N132* (green) variants; 3 mutants for I127*; and a single variant for E131 (pink). The mutants V126K (red), I127V (indigo), A130L (orange), E131D (blue), and N132R (pink) are highlighted with specific data points, as they exhibit Δ*G* values lower than the calculated confidence interval's lower limit. The 95% confidence interval (transparent green band) was estimated from the Δ*G* (MMGBSA) values obtained from four 200 ns replicas of the wild-type (WT) system.

To establish the baseline binding energy of the RgpB/ProRgpB complex that the mutants needed to surpass, four MMGBSA binding energy calculations were performed using independent 200 ns replicas of the wild-type (WT) system. A 95% confidence interval (*α* = 0.05) was calculated from the Δ*G* values, with the lower limit considered as the minimum binding energy threshold for mutant selection. As shown in Fig. 4, at least one mutant from each targeted variant (V126K, I127V, A130L, E131D, and N132R) exhibited a potentially higher affinity for RgpB. In total, 34 mutants were identified with Δ*G* values more negative than the lower confidence limit obtained for the WT replicas (Fig. 4). These results suggest that the wild-type loop conformation may be naturally optimized to facilitate its dissociation in the extracellular environment.

The observed trend is consistent with the statistical distribution analysis, suggesting that it is not an exception but rather a predominant feature within the set of analyzed mutants. The obtained energy values are comparable to those reported in previous studies based on MMGBSA calculations of protein complexes, such as β2-microglobulin/D76Nal, SARS-CoV-2 Main Protease or LFA/SARS-CoV-2 Orf7a dimers, where key residues for dimerization were identified, although structural mutations were not evaluated.^38–40^ The binding free energies calculated in this study by MMGBSA are of the same order of magnitude as those reported for the LL-37/FimA complex by MMPBSA, despite the larger interaction interface involved in the ProRgpB/RgpB complex.^41^ In the RgpB/ProRgpB complex, the free energy distributions show a shift toward more negative values compared to the wild type, with approximately 65.4% (34 mutants) of the variants exhibiting a lower Δ*G*, indicating a higher affinity for RgpB according to the applied criterion. Within this group, the N132* variant stands out, with 81.3% (15 mutant) of its mutants exhibiting a more negative Δ*G* than the WT. However, the inherent variability of the calculations and their accuracy limitations may not be sufficient to support definitive conclusions, even considering the high number of mutants analyzed (52 variants). For this reason, three mutants were selected for a more exhaustive analysis by performing four additional replicas of 200 ns each.

#### 2.1.2 Affinity evaluation of the ProRgpB inhibitory loop variants for RgpB

Δ*G* calculations using MMGBSA provide a rapid approximation for an initial affinity assessment; however, they have limitations associated with inherent methodological errors, making a statistical analysis necessary to discriminate the obtained values and rank them in a statistically reliable manner. To this end, an analysis of variance (ANOVA) followed by Tukey's multiple comparisons test was applied to evaluate whether there were significant differences in the binding energy between RgpB and ProRgpB.

**Fig. 5:**
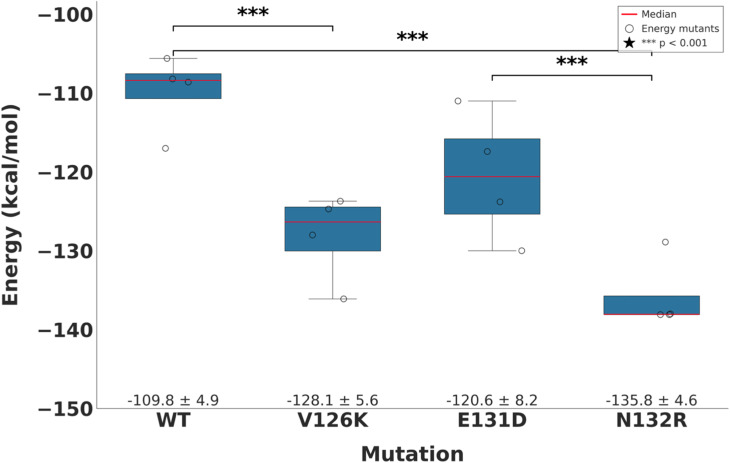
Analysis of variance with Tukey's test for the Δ*G* of the selected inhibitory loop mutants. The Δ*G* (MMGBSA) from four 200 ns simulation replicas of the WT, V126K, E131D, and N132R variants was analyzed using ANOVA followed by Tukey's post-hoc test (*p* = 0.001). Each box represents the distribution of the Δ*G* values for each group, showing the median, quartiles, and individual values (circles). The *X*-axis indicates the confidence interval for the Δ*G* of the four replicas for each variant, including WT. Significant differences between pairs of variants are highlighted with ***, with a black line connecting the compared groups and a red line indicating the means.

Three inhibitory loop mutants (V126K, E131D, and N132R) that exhibited more negative binding energies than the lower confidence limit calculated for the WT (Fig. 4) were selected. With these variants, along with the WT system, additional 200 ns simulations were performed in four independent replicas, from which the Δ*G* was recalculated using MMGBSA. The results obtained (Fig. 5) allow for the comparison of the groups and establish the existence of statistically significant differences between them.

As shown in Fig. 5, the V126K and N132R variants show significant differences in binding Δ*G* values compared to the WT. In contrast, the E131D variant exhibits no significant differences from the WT, which is consistent with the greater dispersion of its Δ*G* values and a less negative average (−120.6 ± 8.2 kcal mol^−1^). However, the Δ*G* of E131D differs significantly from that of N132R, which corresponds to the most negative value among all analyzed variants (−135.8 ± 4.6 kcal mol^−1^).

In the case of the asparagine-to-arginine substitution (N132R), this increases the side chain length, preserves an amino group attached to a planar sp^2^ carbon, and introduces a positive charge. This replacement is less disruptive than the valine-to-lysine substitution (V126K), in which a hydrophobic radical is replaced by a positively charged amino group that is more mobile due to being attached to an sp^3^ carbon. These structural modifications at different positions in the loop could explain the observed changes in Δ*G*; however, the methodology used does not allow for a definitive discrimination between these effects. For instance, in the E131D case, the substitution only increases the chain length while maintaining the carboxylic acid group, implying a minor structural alteration that may be masked by the error of the method.

Taken together, these results suggest that certain positions may constitute viable mutation targets with the potential to significantly increase the affinity of ProRgpB for RgpB. However, to evaluate these improvements more accurately, it is necessary to employ methodologies that reduce the error in Δ*G* calculations.

### 2.2 Calculation of ΔΔ*G* for stabilizing mutations in the RgpB/ProRgpB complex

Alchemical binding free energy (BFE) calculations constitute a thermodynamically rigorous methodology for estimating ligand–protein affinity variations, achieving a balance between precision (∼0.75 kcal mol^−1^), correlation with experimental data, and speed.^42^ Considering this balance of speed and precision, the free energy variations (ΔΔ*G*) between the selected mutations (V126K, E131D, and N132R) and the WT variant were calculated (Table 1). However, these results should be interpreted primarily in a comparative manner rather than as absolute energetic differences for each individual mutant relative to the WT. Since all calculations were performed using the same simulation protocol and thermodynamic framework, the relative trends and differences among mutants are expected to be more reliable than the absolute ΔΔ*G* values themselves, thereby reducing the risk of overinterpreting individual energetic estimations.

**Table 1:** ΔΔ*G* for the selected mutations^a^

	Δ*G* (kcal mol^−1^)		ΔΔ*G* (kcal mol^−1^)
Mutation ProRgpB	RgpB/ProRgpB^1^	ProRgpB^2^	
V126K	−118.83	−107.25	−11.6
E131D	−32.28	−28.40	−3.9
N132R	−156.26	−142.71	−13.6

a 1 complex, 2 ligand.

Considering the BFE calculations, the selected mutations favor the binding of ProRgpB to RgpB, with Δ*G* variations consistent with the results of the ANOVA and Tukey test, particularly for the V126K and N132R variants. These mutations exhibit ΔΔ*G* values approximately four times greater than those observed for E131D, which showed no statistically significant differences in that analysis. However, the greater sensitivity of the BFE method reveals a difference of −3.6 kcal mol^−1^ for D131E, which, although smaller than that of the other variants, remains relevant for complex stability.

In the case of V126K and N132R, the ΔΔ*G* values exceed 10 kcal mol^−1^, which may partly reflect an overestimation of the energetic variation due to the magnitude of the structural change introduced. These values are comparable to those reported for mutants at the tetramer interface of human transthyretin, where the His88Phe mutation shows a ΔΔ*G* of approximately 10 kcal mol^−1^, like our calculations, or to the ABE8e D119N mutant in complex with ABE7.10, which exhibits a value of around −7.8 kcal mol^−1^.^43,44^ However, unlike our results, which are based on a model specifically selected to undergo dissociation, fewer variants display increased affinity. In the case of our results, according to the relationship:

1 ΔΔ*G* = *RT* ln(*K*_i_^mut^/*K*_i_^WT^),

with *R* = 0.001987 kcal mol^−1^ K^−1^ and *T* = 298 K, a ΔΔ*G* > 10 kcal mol^−1^ implies an increase in affinity greater than 20 million-fold, equivalent to a shift in *K*_i_ from the mM to the nM range, representing a substantial improvement in binding.

However, although *K*_i_ values in the nM range have been reported for ProRgpB, these correspond to assays with synthetic substrates of lower affinity than natural substrates, which could lead to an overestimation of these values.^45^ In this context, the results obtained from the BFE calculations would be more consistent with an affinity increase of this magnitude, while always considering potential experimental bias. In contrast, for the E131D mutation, the Δ*G* variation predicts an affinity increase of nearly 700-fold, constituting a relevant change that is much more in line with available experimental data.

Collectively, although the BFE calculations may overestimate affinity, especially for the V126K and N132R variants, the results constitute a relevant finding that justifies their validation through future experimental studies.

## 3. Methods

### 3.1 Model construction

Starting from the crystallographic coordinates of the RgpB/ProRgpB complex^24^(PDB ID: 4IEF, resolution 2.3 Å), a complete structural model was generated using SwissModel, which enabled the reconstruction of the unresolved region of the ProRgpB protein^46^ (Fig. S4). To model the missing segment, corresponding to residues Ser185–Thr191 of ProRgpB, the full-length sequence of this protein was used, with the crystallographic structure deposited in the Protein Data Bank (PDB ID 4IEF) serving as the template through the ‘User Template’ option of the SwissModel online platform (Fig. S4C). The final model comprises residues 24–423 of RgpB and 9–207 of ProRgpB,^47^ according to the sequence deposited in UniProt (accession code P95493).

The ionization states of the residues were calculated using the H++ server, and the structure was parameterized using the ff14SB force field. Subsequently, the complex was solvated in a box of explicit water represented by the TIP3P model, and charges were neutralized by adding Na^+^ or Cl^−^ ions.^48,49^

The inhibitory loop region of ProRgpB was aligned with the corresponding region of ProKgp (Fig. S1) to identify fully conserved residues, residues conserving chemical properties, and non-conserved residues (Fig. 1D). This analysis was used to define the mutational strategy for rational design. Conserved residues (S128, R129, and P133) were preserved due to their potential structural and functional relevance in RgpB/ProRgpB binding and inhibition (Fig. S6b). Residues conserving side-chain chemical properties (I127 and E131) were subjected to conservative substitutions, generating the mutants I127A, I127L, I127V, and E131D. In contrast, residues V126, A130, and N132, which did not conserve side-chain chemical properties relative to ProKgp, were mutated to selected amino acids while avoiding glycine, proline, and tryptophan to minimize structural perturbation of the inhibitory loop. The generated mutants were V126A, V126D, V126E, V126F, V126I, V126K, V126R, V126Y, V126C, V126H, V126L, V126M, V126Q, V126S, V126T, and V126N; I127A, I127L, and I127V; A130F, A130L, A130N, A130Q, A130Y, A130C, A130D, A130E, A130H, A130K, A130M, A130R, A130S, A130T, A130V, and A130I; E131D; and N132A, N132D, N132K, N132M, N132R, N132Q, N132C, N132E, N132F, N132H, N132I, N132L, N132S, N132T, N132V, and N132Y. In total, 52 inhibitory loop mutants were generated and subsequently evaluated for RgpB/ProRgpB complex stability to estimate their potential affinity for RgpB.

**Fig. 6:**
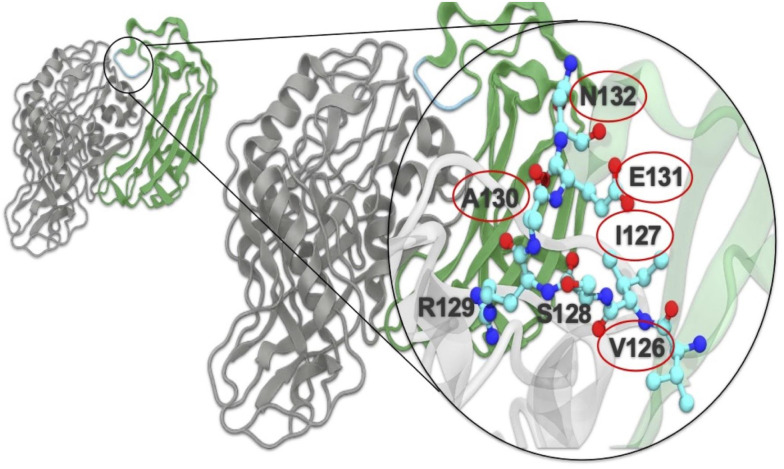
Mutation sites. The RgpB/ProRgpB complex is shown together with a magnified view of the inhibitory loop region, highlighting the mutated residues. Carbon atoms are shown in cyan, nitrogen atoms in blue, and oxygen atoms in red.

From the native structure, a total of 52 mutants were generated using the LEaP module of AmberTools by directly editing the coordinates of the WT model.^50^ These variants included 16 substitutions for residue N132, one for E131 (E131D), 16 for A130, three for I127, and 16 for V126, selected according to the positions identified in Fig. 6.

### 3.2 Molecular dynamics simulation

Molecular dynamics simulations were performed using AMBER24-GPU following an established protocol.^49,51^ Initially, the system underwent a stage of minimization and structural relaxation. This included 2000 steps of minimization on the water molecules, followed by a simulation in the NPT ensemble (300 K, 1000 ps) with harmonic restraints of 10 kcal mol^−1^ Å^−2^ applied to the polypeptide chain. Subsequently, a global minimization of the structure was conducted using 6500 steps of the conjugate gradient algorithm.^52^

The minimized systems were progressively heated to 300 K with restraints on the protein backbone over 0.5 ns, and then equilibrated in two phases: 0.5 ns with restraints and 5 ns without restraints in the NVT ensemble at 300 K. The final production simulation was extended for 50 or 200 ns for each system.

Throughout the simulations, the equations of motion were integrated with a 2 fs time step in the NPT ensemble at 1 atm. The SHAKE algorithm was applied to constrain bonds involving hydrogen atoms. A cutoff of 12 Å was used for van der Waals interactions. Temperature control was maintained using the Langevin thermostat (300 K, collision frequency of 2 ps^−1^), and pressure was regulated with the Berendsen barostat (1 atm). Long-range electrostatic interactions were handled using the Particle Mesh Ewald (PME) method.^53^ Trajectory data were saved every 0.025 ps, and subsequent trajectory analysis and visualization were performed using CPPTRAJ and the VMD software.^54^

### 3.3 Principal component analysis (PCA)

Principal Component Analysis (PCA) was employed as a dimensionality reduction method to characterize the most relevant collective motion modes from the molecular dynamic's trajectories.^55^ This method transforms the high-dimensional dataset—originating from atomic coordinates—into a reduced space that retains most of the system's variability. Specifically, PCA identifies the directions of maximum variance in the conformational space, projecting the energy landscape onto its first two principal components (PC1 and PC2).

The analysis was applied to an extended 1.5 µs simulation and a set of 52 independent 50 ns simulations, aiming to comprehensively capture the conformational diversity accessible to the protein complex. From the joint projection of the trajectories in the space defined by the first two principal components (PC1 *vs.* PC2), the centroid corresponding to the most populated conformational distribution was identified. The structure associated with this centroid was extracted directly from the trajectories and selected for subsequent structural analysis, thereby representing the most characteristic average conformation of the system under study.

### 3.4 MM/GBSA binding energy calculation

The MM/GBSA (Molecular Mechanics Generalized Born Surface Area) method was employed to estimate the binding free energy of the RgpB/ProRgpB complex for the different inhibitory loop variants.^35,56,57^ Using molecular dynamics simulations of 50 ns or 200 ns, the last 40 ns of each trajectory were analyzed, after removing explicit water molecules and ions. The energy calculations were performed separately for three components of each system: the RgpB protein alone, the ProRgpB inhibitory peptide alone, and the complete complex.

The total free energy (Δ*G*_tot_) for each entity was calculated using the following equation:

2 Δ*G*_tot_ ∼ EMM + *G*_solv_

where:

EMM is the molecular mechanics energy, which includes contributions from bonded (bonds, angles, and dihedrals) and non-bonded (electrostatic and Lennard–Jones) terms. The entropic contribution (−*T*Δ*S*) was omitted from the equation, as commonly done in previous studies, mainly due to its high computational cost and the convergence problems and large statistical uncertainties typically observed in large and flexible biomolecular systems.

*G*_solv_ represents the solvation energy, composed of polar and non-polar components.

The EMM and *G*_solv_ terms were calculated using AMBER-GPU with the generalized born implicit solvation model.^56,58^ The non-polar component of *G*_solv_ was estimated as a linear function of the solvent accessible surface area (SASA), calculated with a probe radius of 1.4 Å.

Finally, the binding free energy (Δ*G*_bind_) (eqn (3)) was obtained as:

3 Δ*G*_bind_ = *G*_tot(RgpB/ProRgpB)_ − *G*_tot(RgpB)_ − *G*_tot(ProRgpB)_

where each *G*_tot_ value corresponds to the average calculated over the analyzed frames from the production phase of the simulation.

### 3.5 Statistical analysis

To compare the binding free energy (Δ*G*_bind_) estimated by MM/GBSA among the different variants of the RgpB/ProRgpB complex (WT, V126K, E131D, N132R), the following statistical procedure was performed.

Initially, the precision and variability of the estimates obtained from 50 ns simulations were evaluated using box and whisker plots, constructed from 52 Δ*G*_bind_ estimates calculated for each variant. The stability of these estimates was contrasted against a 95% confidence interval derived from the binding energies of the WT variant, obtained from four independent 200 ns simulation replicates.

Subsequently, the multiple comparison statistical analysis was performed directly on the set of four 200 ns replicates for each variant, which was considered the most robust dataset. Assuming the existence of significant differences among the groups, a post-hoc Tukey HSD (Honestly Significant Difference) test was directly applied to compare all pairs of variants against each other, controlling the family-wise error rate and thereby determining which specific differences were significant.^59^ All analyses were performed using a custom-developed Python script, and a *p*-value < 0.05 was considered the threshold for statistical significance.

### 3.6 Calculation of binding free energies using thermodynamic integration

The change in binding free energy (ΔΔ*G*) for the selected mutations in the ProRgpB inhibitory loop (V126K, E131D, and N132R) relative to the wild-type (WT) complex was calculated using the thermodynamic integration (TI) method.^36^ The alchemical transformations between each variant and the reference system were modeled using a unified protocol in which electrostatic and van der Waals (vdW) interactions were scaled simultaneously using softcore potentials. These interactions were gradually modified *via* a coupling parameter (*λ*), which transforms the system from the initial state (WT, *λ* = 0) to the final state (mutant, *λ* = 1), utilizing a total of 13 discrete windows (*λ* = 0.0, 0.05, 0.1, 0.2, 0.3, 0.4, 0.5, 0.6, 0.7, 0.8, 0.9, 0.95, 1.0).^60^

**Fig. 7:**
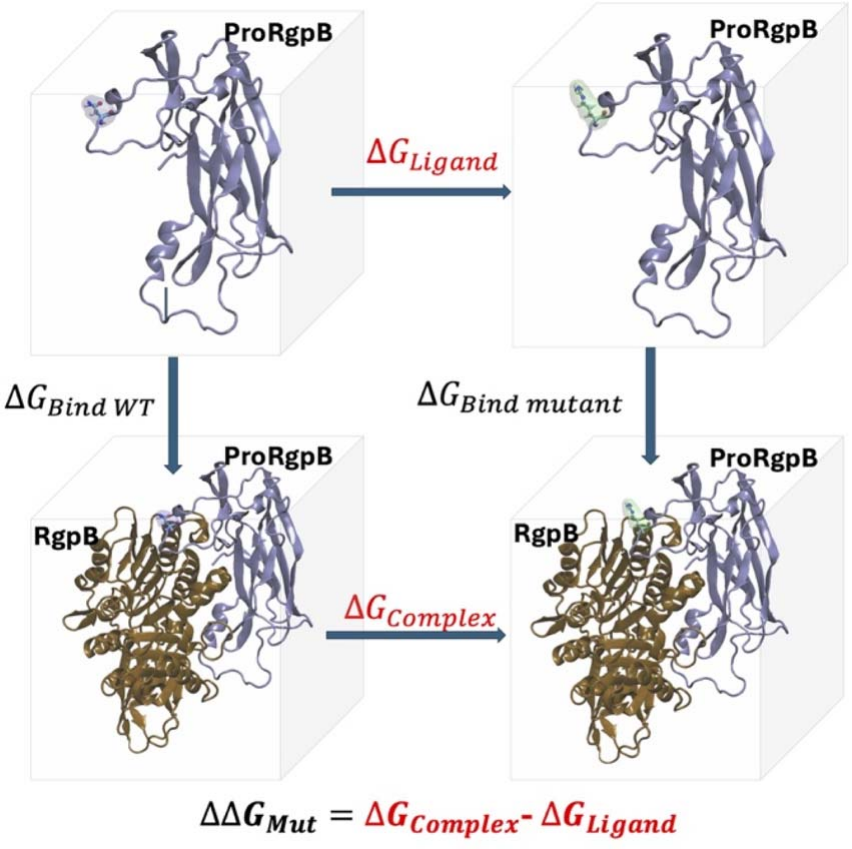
Thermodynamic cycle for the calculation of relative binding free energies (ΔΔ*G*). The cycle illustrates the alchemical transformation between the wild-type (WT, residue in blue) and a mutated variant (residue in green) of the inhibitory peptide ProRgpB, both in the free state (top path) and in complex with the RgpB enzyme (bottom path, depicted in indigo). The relative binding free energy change for each mutation (ΔΔ*G*_Mut_) is calculated as the difference between the free energy change of the mutation in the complex (Δ*G*_Complex_) and in the free ligand (Δ*G*_Ligand_). This thermodynamic cycle allows for the quantification of the energetic effect of each mutation on the stability of the RgpB/ProRgpB complex.

The Δ*G* values for each transformation were obtained by numerical integration of 〈∂*U*/∂*λ*〉 across the *λ* windows, applying the alchemical extrapolation method and the trapezoidal rule. Long-range electrostatic interactions were treated with the PME method, and vdW interactions were calculated with a cutoff radius of 9 Å.^53^ The second-order soft-core step potential was implemented with *α* = 0.5 and *β* = 12 Å^2^ (ref. 60). Each *λ* window underwent an energy minimization step, followed by 5 ns of equilibration in the NPT ensemble and 15 ns of production. The TI simulations were performed with the pmemd.cuda module of AMBER20 in the NPT ensemble at 300 K and 1 atm, using an isotropic Monte Carlo barostat with a relaxation time of 2.0 ps. All simulations used a 1 fs timestep with hydrogen mass repartitioning (HMR). The relative binding free energy (ΔΔ*G*) for each mutation was determined by applying the thermodynamic cycle shown in Fig. 7, taking the last 5 ns of production after confirming the convergence of the windows.

## 4. Conclusions

The computational model generated from the crystal structure of the RgpB/ProRgpB complex showed high stability during ∼1.5 µs of molecular dynamics simulation, with fluctuations of less than 3 Å in the carbon backbone (Fig. 1A) and consistent RMSD values for both the complex and each individual component (Fig. S2). The highest fluctuations were localized in regions distant from the inhibitory loop and the catalytic site, particularly near PRO88 of RgpB and THR203 of ProRgpB (Fig. 1B and C). Analysis of the last 0.8 µs allowed for the identification of a representative centroid conformation obtained *via* principal component analysis (Fig. S2), which was used to design 52 mutants of the ProRgpB inhibitory loop.

Mutations were defined based on the degree of residue conservation in the inhibitory domain of RgpB and Kgp, classifying them into: (i) fully conserved, which were not modified; (ii) conserved in their chemical nature, substituted with amino acids of similar properties; and (iii) non-conserved, where all substitutions except glycine and tryptophan were tested.

Fifty-nanosecond molecular dynamics simulations enabled the calculation of binding energies using MMGBSA and the analysis of the structural effects of each mutation. The results show that after the first 10 ns, the structures stabilize (Fig. 2 and 3), maintaining high stability in both the catalytic site and the inhibitory loop. Statistical analysis of the binding energies indicated that all evaluated positions contain mutants with higher affinity than the wild-type protein, with position N132* standing out, having over 80% of its mutants more stable than WT (Fig. 4).

For the V126K, E131D, and N132R mutations, additional 200 ns simulations (four replicas each) followed by analysis of variance and Tukey's test revealed significant differences for V126K and N132R (Fig. 5). Alchemical binding free energy (BFE) calculations confirmed that all three mutations favor complex formation, with V126K and N132R being the most prominent, showing ΔΔ*G* variations greater than −10 kcal mol^−1^ (Table 1).

Collectively, these results establish a protocol for evaluating how point mutations affect protein–protein interactions and provide a foundation for the design of *in vitro* assays aimed at developing inhibitors based on the binding of ProRgpB to RgpB.

## Author contributions

Sebastián Tapia: methodology, research, and calculations. Olimpo García-Beltrán: writing (original draft), research. Denisse Bravo: draft review and visualization. Daniel Bustos: validation, formal analysis. Osvaldo Yañez: validation, methodology, research. Manuel Osorio: writing (review and editing), validation, supervision, resources, project management, conceptualization.

## Conflicts of interest

There are no conflicts to declare.

## Supplementary Material

RA-OLF-D6RA01702A-s001

## Data Availability

A data this database contains the output files from the 01_PROD and 02_REP simulations, corresponding to the molecular dynamics simulations performed with the AMBER software. The 01_PROD directory includes the results from the 52 production simulations, while the 02_REP directory contains one of the replicas. The 03_TI directory contains the output files from the TI calculations for a mutation, considering both the complex (RgpB/ProRgpB) and the ligand (ProRgpB). The supporting information is available at Zenodo: https://doi.org/10.5281/zenodo.17466665. Supplementary information (SI) is available. See DOI: https://doi.org/10.1039/d6ra01702a.
