# Supplementary material for: Mutagenesis and free energy calculations to optimize the ProRgpB inhibitor loop and identify variants with higher affinity for Porphyromonas gingivalis RgpB
Source: RSC Adv. 2026 Jul 14;16(37):40226–36. doi: 10.1039/d6ra01702a (PMC13366090; doi:10.1039/d6ra01702a)
Supplement: Supplementary file 1 [file RA-016-D6RA01702A-s001.pdf]

Supplementary Information (SI) for RSC Advances.
This journal is © The Royal Society of Chemistry 2026

Mutagenesis and free energy calculations to optimize the ProRgpB inhibitor loop and identify variants with higher affinity for *Porphyromonas gingivalis* RgpB.

Sebastián Tapia[1], Osvaldo Yañez[2], Olimpo García-Beltrán[3,4], Daniel Bustos[6], Denisse Bravo[7] y Manuel I. Osorio[5,7].

1. Computational & Quantum Enzyme Modeling Lab. Facultad de Odontología, Universidad Andres Bello, Santiago, Chile, Echaurren 237, Santiago 8370133, Chile.

2.Centro de Modelación Ambiental y Dinámica de Sistemas (CEMADIS), Facultad de Ingeniería y Negocios, Universidad de Las Américas, Santiago, Chile

3. Facultad de Ciencias Naturales y Matemáticas, Universidad de Ibagué, Carrera 22 Calle 67, Ibagué 730002, Colombia

4. Centro Integrativo de Biología y Química Aplicada (CIBQA), Universidad Bernardo O'Higgins, General Gana 1702, Santiago 8370854, Chile

5. Facultad de Medicina, Centro de Investigación Biomédica, Universidad Diego Portales, Ejército 141, Santiago 8320000, Chile

6. Laboratorio de Bioinformática y Química Computacional, Departamento de Medicina Traslacional, Facultad de Medicina, Universidad Católica del Maule, Talca 3480094, Chile. dbustos@ucm.cl https://orcid.org/0000-0002-2136-2305

7. Facultad de Odontología, Universidad Andres Bello, Santiago Chile, Echaurren 237, Santiago 8370133, Chile.

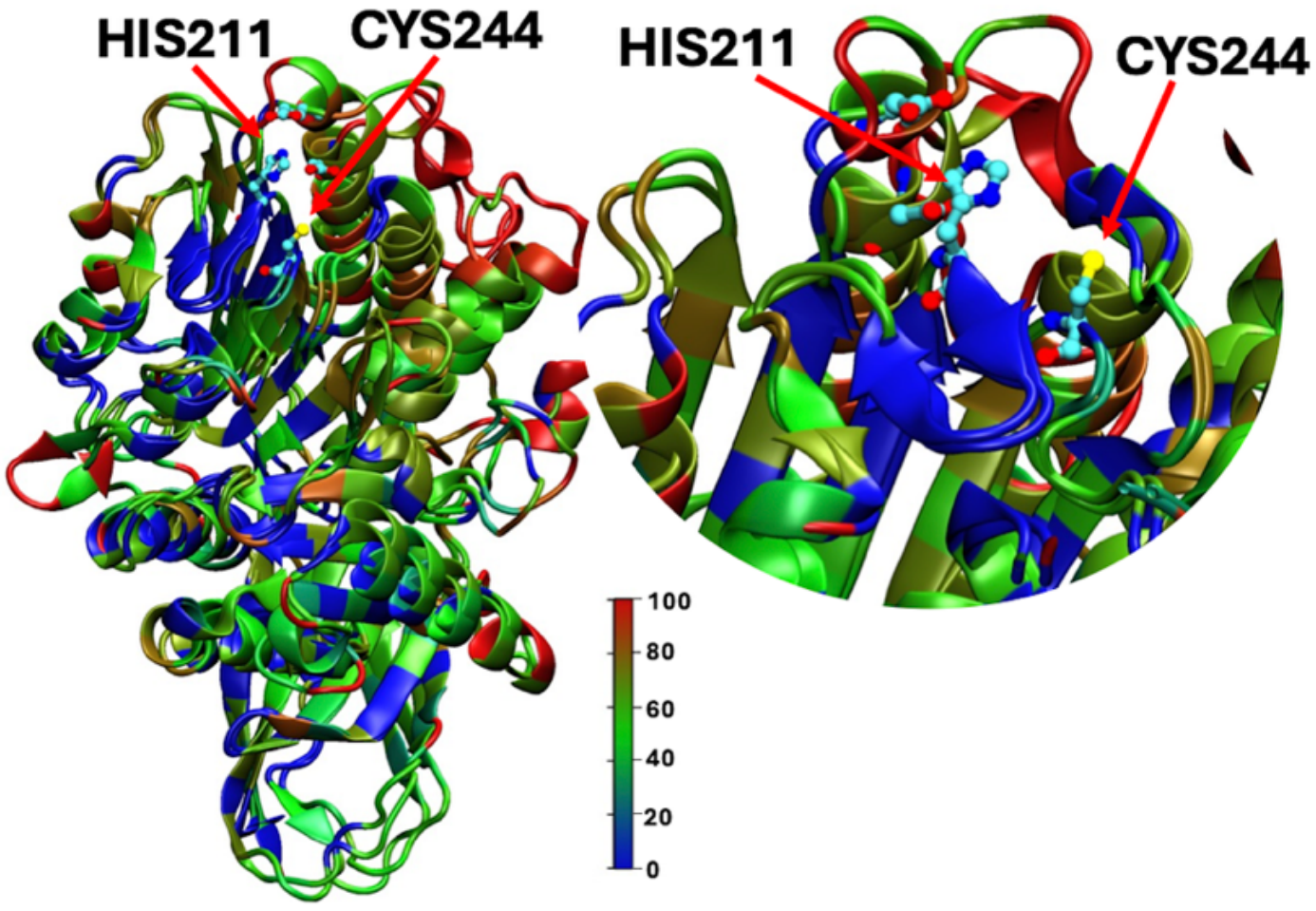

Figure S1. Structural similarity of gingipains RgpB and KgP. The crystal structures of RgpB (PDB code 1CVR) and KgP (PDB code 4TKX) were aligned using the VMD program, and the residues were colored according to root mean square deviation (RMSD) between the structures, the 100% is 2.6 Å. The catalytic residues HIS 211 and CYS244 are highlighted as they are located at the binding site of the Prodomain inhibitor loop.

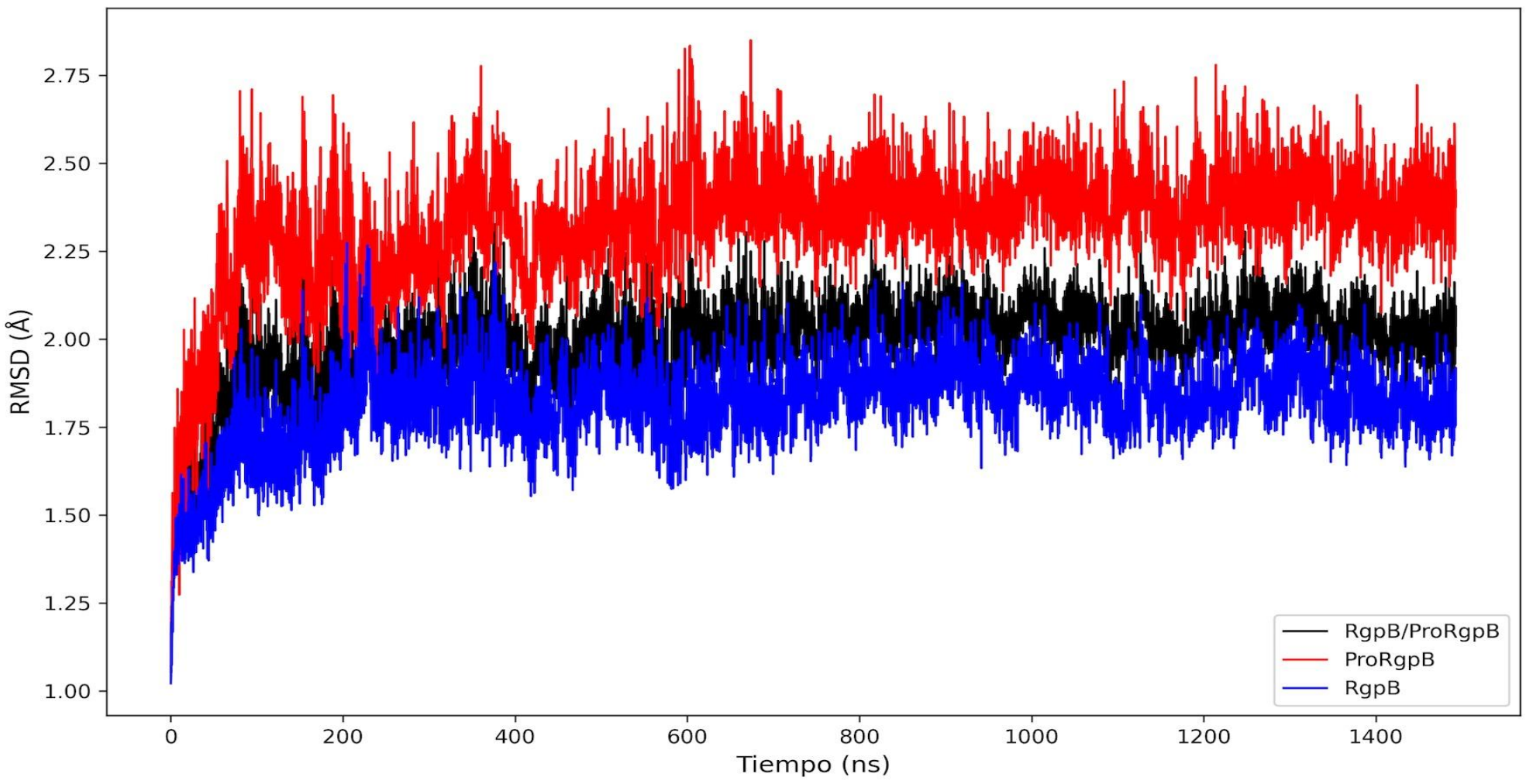

Figure S2. Structural stabilization of the RgpB/ProRgpB complex. A 1500 ns molecular dynamics simulation in an explicit water box was analyzed to calculate the RMSD of the complex (black line), the RgpB enzyme (blue line), and the ProRgpB prodomain (red line).

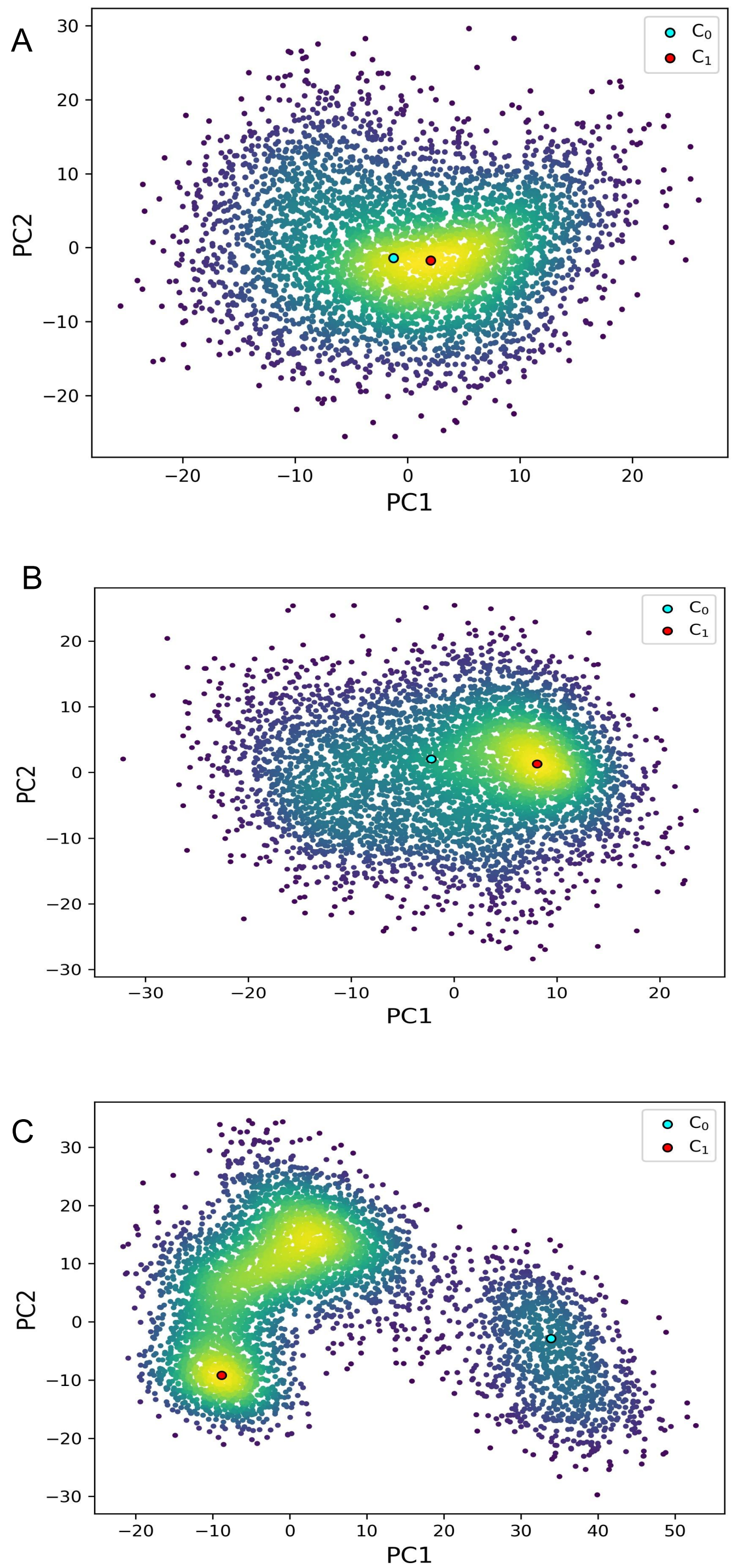

Figure S3. Principal component analysis (PCA) of the 1500 ns molecular dynamics simulation of the RgpB/ProRgpB complex. The entire trajectory (C), the last 1000 ns (B), and the last 800 ns (A) of the simulation were analyzed. The two main clusters identified from the structural positions during the simulation are indicated by light blue (C_0) and red circles (C_1), respectively. The representative centroid of each cluster is shown in yellow.

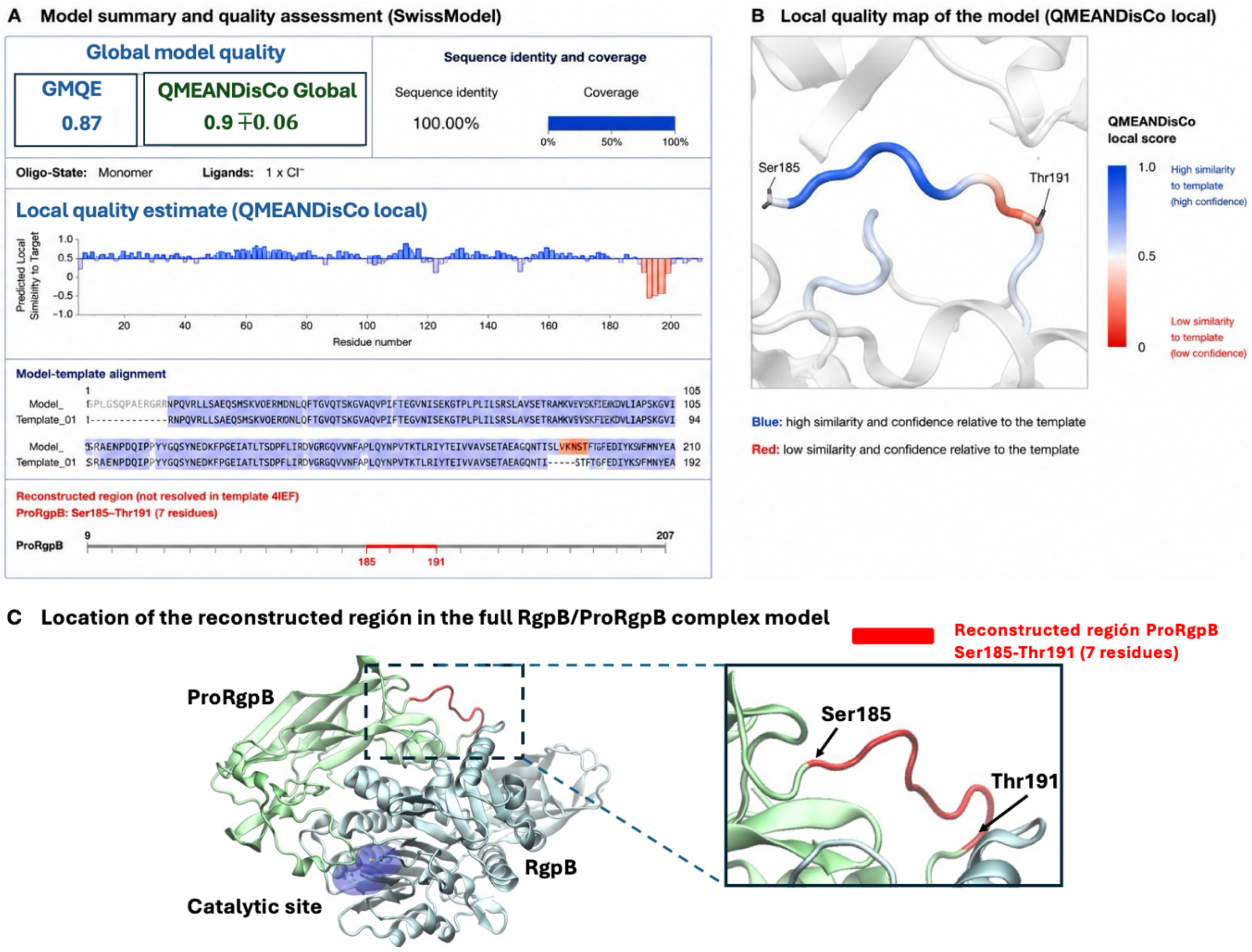

Figure S4. Structural quality assessment of the ProRgpB model for the 185–191 loop region. A complete structural model of the ProRgpB zymogen was generated using the SWISS-MODEL online platform, employing the crystal structure 4IEF as template. In (A), the alignment between the sequence of the structure used as template (Template_01) and the target sequence corresponding to ProRgpB (Model) is shown, together with the global and local quality assessment of the model. Global quality was estimated using QMEAN (Qualitative Model Energy ANalysis), a composite scoring function based on statistical potentials and structural agreement terms, whereas local per-residue quality was evaluated using QMEANDisCo, which incorporates distance constraints derived from homologous structures to estimate structural reliability. Quality scores are presented in the range [0–1], where values close to 1 indicate higher structural reliability. In (B), a close-up view of the modeled loop region comprising residues 185–191 is shown. In (C), the location of this loop relative to the catalytic site of RgpB (blue region) within the complete ProRgpB model is presented, together with its spatial arrangement with respect to the inhibitory loop of ProRgpB.

Table S1. Hydrogen bonds classified by occupancy range (He et al., 2024) for WT and mutants V126K, E131D, N132R. Occupancy = fraction of simulation time the bond persists. Numbers indicate counts of distinct H-bonds in each category. Total bonds per system: WT = 28,842; V126K = 28,919; E131D = 28,682; N132R = 28,657.

	**Hydrogen bond Occupancy**			
	0%-10%	10%-30%	30%-50%	50%-100%
**WT**	27956	335	234	317
**V126K**	28024	334	231	330
**E131D**	27818	316	230	318
**N132R**	27778	312	246	321

The analysis of hydrogen bonds in the 200 ns simulations (four replicates per system) shows that, in all cases, the vast majority of interactions are transient (occupancy below 50%), while only a small fraction (between 317 and 330 bonds per system) remain stable for more than 50% of the simulation time. This phenomenon, in which an extensive and dynamic network of transient hydrogen bonds coexists with a reduced number of stable interactions, has also been observed in other computationally modeled systems (He et al., 2024). The V126K, E131D, and N132R mutants exhibit a slightly higher number of strong hydrogen bonds compared to the wild-type (WT) protein, which is qualitatively consistent with their negative ΔΔG values (higher affinity). However, no linear correlation is observed between the number of strong hydrogen bonds and the magnitude of ΔΔG, suggesting that the additional stabilization does not depend solely on the number of permanent hydrogen bonds, but also on their geometric quality (short distances and angles close to 180°) and the cooperativity of the weak interaction network.

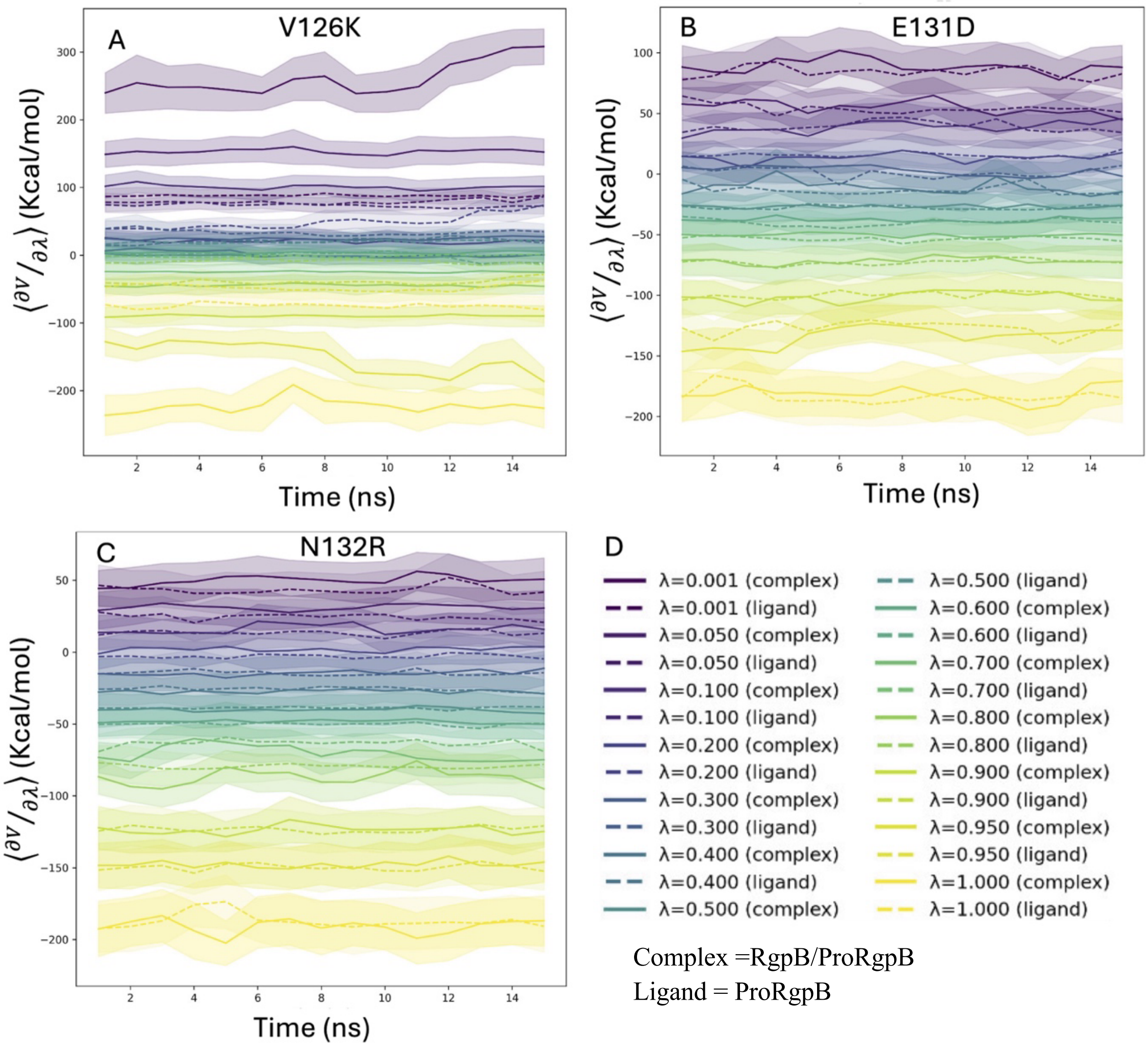

Figure S5. Convergence analysis of thermodynamic integration simulations for the V126K (A), E131D (B), and N132R (C) variants. The ensemble averages of the alchemical derivative, ⟨∂V/∂λ⟩, are shown as a function of simulation time for the protein–ligand complex (solid lines) and the ligand in aqueous solution (dashed lines) at each λ window. Shaded areas correspond to the standard deviation obtained from block averaging. The absence of systematic drifts and the overall stability of the gradients over time indicate adequate sampling and convergence of the free-energy calculations. Panel (D) displays the λ schedule and corresponding color scheme.

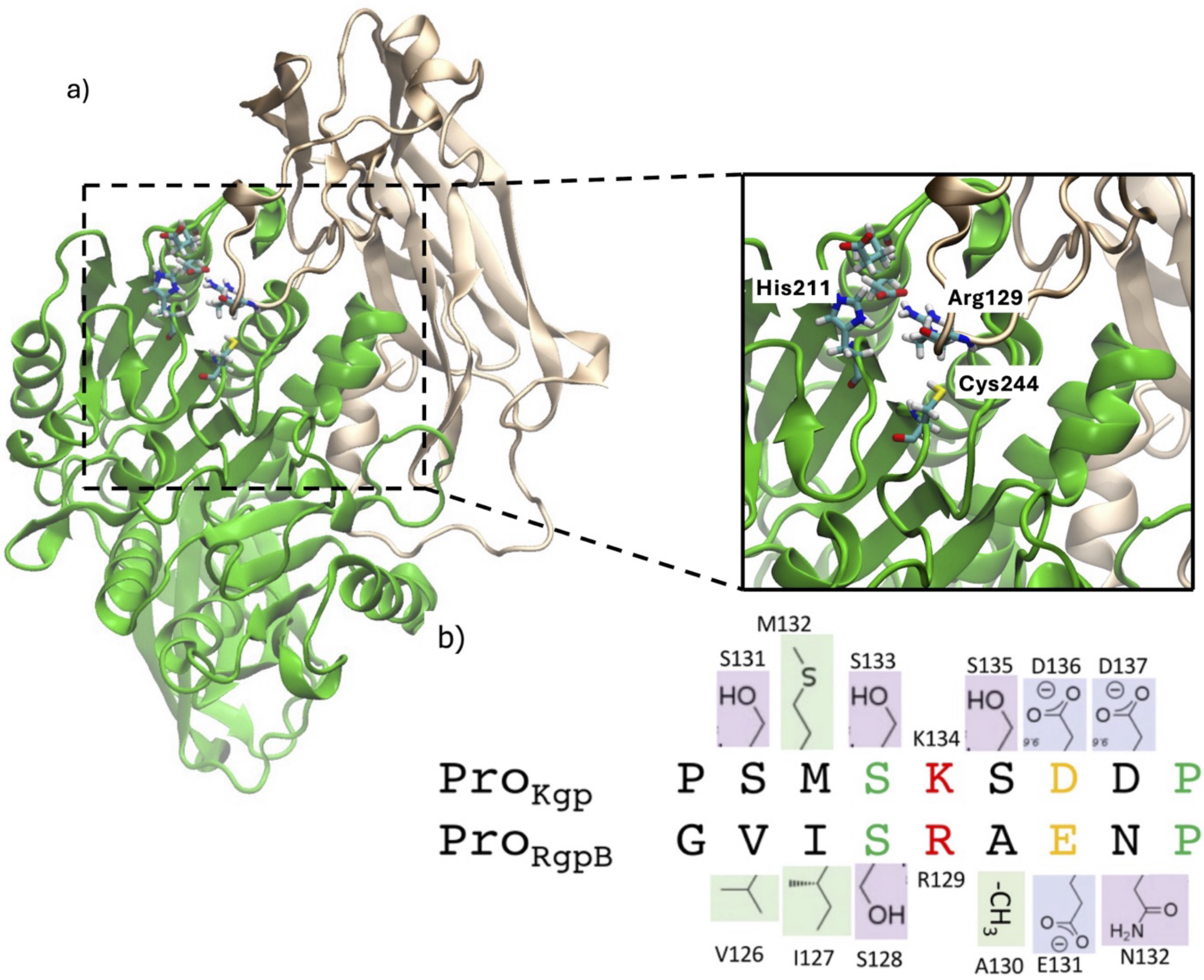

Figure S6. Position of the inhibitory loop within the catalytic site of RgpB. The structure of the RgpB/ProRgpB complex is shown together with a magnified view of the catalytic site, highlighting Arg129 of the inhibitory loop, which is recognized by the enzyme specificity pocket, as well as the catalytic residues Cys144 and His211. (b) Sequence alignment of the zymogen loops from Kgp (ProKgp) and RgpB (ProRgpB), highlighting the degree of conservation between both proteins. Residues shown in green are fully conserved, residues in yellow correspond to conservative substitutions that preserve similar physicochemical properties, and residues in black represent non-conserved positions.

Table S2. MM-GBSA binding energies (kcal/mol) from a single 50 ns simulation replica (seed -1) for point mutations at positions 126, 127, 130, 131, and 132 of ProRgpB. More negative values denote stronger binding. The N132R mutant shows the most favorable energy in the series.

**Mutant**	**MMGBSA**	**Mutant**	**MMGBSA**	**Mutant**	**MMGBSA**
**V126**		**I127**		**A130**	
V126A	-109.7	I127A	-123.6	A130F	-125.2
V126D	-126.6	I127L	-105.0	A130L	-136.6
V126E	-109.2	I127V	-132.4	A130N	-127.9
V126F	-120.3			A130Q	-121.6
V126I	-112.1	**E131**		A130Y	-123.8

V126K	-125.1	E131D	-133.1	A130C	-104.0
V126R	-135.7			A130D	-108.2
V126Y	-119.4	**N132**		A130E	-124.1
V126C	-121.7	N132A	-116.8	A130H	-116.4
V126H	-121.1	N132D	-130.5	A130K	-120.4
V126L	-117.7	N132K	-132.7	A130M	-127.1
V126M	-115.4	N132M	-130.5	A130R	-117.8
V126Q	-116.5	N132R	-144.4	A130S	-136.2
V126S	-104.6	N132Q	-133.7	A130T	-111.1
V126T	-119.3	N132C	-125.0	A130V	-107.9
V126N	-109.9	N132E	-124.1	A130I	-113.5
		N132F	-122.2		
		N132H	-119.6		
		N132I	-113.0		
		N132L	-115.2		
		N132S	-120.6		
		N132T	-118.8		
		N132V	-127.4		
		N132Y	-128.4		

Table S3. MM-GBSA binding energies (kcal/mol) of ProRgpB/RgpB complexes for wild-type (WT) and the indicated mutants. Values were obtained from 100 ns molecular dynamics simulations (four independent replicates using seeds -1, 40, 100, and 1000). For each system, individual replica energies, mean, and standard deviation (SD) are shown. More negative values indicate higher binding affinity.

	**Seeds**				
**Mutant**	**Ig = -1**	**Ig = 40**	**Ig = 100**	**Ig = 1000**	**Mean +/- SD**
**WT**	-105.6	-117	-108.2	-108.6	-109.9 +/- 4.7
**N132R**	-138.2	-128.9	-138.0	-138.2	-135.8 +/- 4.1
**E1231D**	-130.0	-111.0	-123.8	-117.4	-120.6 +/- 7.4
**V126K**	-136.1	-124.7	-123.7	-128.0	-128.1 +/- 5.4
**A130L**	-136.6	-119.8	-134.9	-119.1	-127.6 +/- 8.5
**I127V**	-132.4	-126.9	-104.1	-106.7	-117.5 +/-12.9
